# Professional Signs and Advertisements

**Published:** 1862-08

**Authors:** 


### PROFESSIONAL SIGNS AND ADVERTISEMENTS.

While it is necessary for the true professional man to avoid even the appearance of quackery, it is possible that he may do himself and the public injustice, by mistaken notions, or over-squeamishness, on the subject of empiricism. Of course he must not advertise as the quack does; but it is not to be inferred from this, that it savors of quackery for him to advertise at all. And if he has a right to advertise at all, he has a right to do so to whatever extent he may see proper. It is not the extent, but the kind of advertising that distinguishes the quack from the professional man. When a man has announced himself, in all the respectable advertising mediums of the land, as "JOHN JONES, DENTIST, Number 21, Fourth Street," he has done nothing inconsistent with the highest standard of professional honor; but he has manifested a degree of energy and enterprise that is likely to be duly rewarded. But if his announcement is, "Joy to the suffering!! The world-renowned Professor John Jones, T.P., P.P., is most happy to be able to announce to the afflicted world that he has returned to his native country, after long years of successful practice among the Royal Families of Africa——," it is fair to conclude that the ponderous initials stand for *Professional Tinker*, and *Pocket Picker*, in short, that the said John Jones is a quack.

The same general principle applies to office-signs. The man who announces his name and business on a neat sign, and places it conspicuously before the public, is only doing justice to himself and his patrons. What right has he to subject his friends to the humiliating necessity of going up and down the highways, inquiring of policemen or neighbors the way to his door? Our patrons seek us on account of their misfortunes and sufferings. Shall we force them to proclaim these, by their anxious inquiries for the dental office? It is not kind to do so. Nor is it modest. The man who does not signify his name and place of business says, as plainly as actions can say, that others may resort to such devices if they see fit, they perhaps need them. *I* have no occasion to resort to them, *my* reputation is such that the public will seek me out, or *my* pecuniary circumstances enable me to rest on my professional dignity till business finds me. And if these assumptions are true, no one can seriously object to his course; but for him to imagine himself the pink of modesty on account of it, is slightly objectionable.

During a ramble in a strange city, we came to a row of houses occupied by the several branches of the Bunker family. On the door-plates were J. Bunker, Thos. Bunker, Wm. Bunker, and BUNKER. Ah! thought we, that's where Bunker *himself* lives. If you have business with Jack, or Tom, or Bill Bunker, you can find him at home; but if you wish to see BUNKER HIMSELF, call at this door. It did not surprise us to learn, from a friend' that the last named was the only snob of the four brothers.

But, on the other hand, the man who sets forth himself and business by outlandish, barbarous signs, such as grinning skeletons, enormous images of monstrous teeth, or formidable pictures of more formidable forceps and cant-hooks, is a quack, and a very disgusting one at that. W.

